# Contribution of Distinct Homeodomain DNA Binding Specificities to *Drosophila* Embryonic Mesodermal Cell-Specific Gene Expression Programs

**DOI:** 10.1371/journal.pone.0069385

**Published:** 2013-07-26

**Authors:** Brian W. Busser, Stephen S. Gisselbrecht, Leila Shokri, Terese R. Tansey, Caitlin E. Gamble, Martha L. Bulyk, Alan M. Michelson

**Affiliations:** 1 Laboratory of Developmental Systems Biology, National Heart Lung and Blood Institute, National Institutes of Health, Bethesda, Maryland, United States of America; 2 Division of Genetics, Department of Medicine, Brigham and Women’s Hospital and Harvard Medical School, Boston, Massachusetts, United States of America; 3 Department of Pathology, Brigham and Women’s Hospital and Harvard Medical School, Boston, Massachusetts, United States of America; 4 Harvard-MIT Division of Health Sciences and Technology (HST), Harvard Medical School, Boston, Massachusetts, United States of America; Université Paris-Diderot, France

## Abstract

Homeodomain (HD) proteins are a large family of evolutionarily conserved transcription factors (TFs) having diverse developmental functions, often acting within the same cell types, yet many members of this family paradoxically recognize similar DNA sequences. Thus, with multiple family members having the potential to recognize the same DNA sequences in *cis*-regulatory elements, it is difficult to ascertain the role of an individual HD or a subclass of HDs in mediating a particular developmental function. To investigate this problem, we focused our studies on the *Drosophila* embryonic mesoderm where HD TFs are required to establish not only segmental identities (such as the Hox TFs), but also tissue and cell fate specification and differentiation (such as the NK-2 HDs, Six HDs and identity HDs (I-HDs)). Here we utilized the complete spectrum of DNA binding specificities determined by protein binding microarrays (PBMs) for a diverse collection of HDs to modify the nucleotide sequences of numerous mesodermal enhancers to be recognized by either no or a single subclass of HDs, and subsequently assayed the consequences of these changes on enhancer function in transgenic reporter assays. These studies show that individual mesodermal enhancers receive separate transcriptional input from both I–HD and Hox subclasses of HDs. In addition, we demonstrate that enhancers regulating upstream components of the mesodermal regulatory network are targeted by the Six class of HDs. Finally, we establish the necessity of NK-2 HD binding sequences to activate gene expression in multiple mesodermal tissues, supporting a potential role for the NK-2 HD TF Tinman (Tin) as a pioneer factor that cooperates with other factors to regulate cell-specific gene expression programs. Collectively, these results underscore the critical role played by HDs of multiple subclasses in inducing the unique genetic programs of individual mesodermal cells, and in coordinating the gene regulatory networks directing mesoderm development.

## Introduction

The precise spatiotemporal control of gene expression is central to the proper restriction of cell fates and for insuring the accuracy of cellular differentiation, essential steps that occur during development [1,2]. This process is orchestrated through enhancers, regions of noncoding DNA that are bound by sequence-specific DNA binding transcription factors (TFs) that target short DNA sequence motifs. The circuitry of TFs and enhancers comprises a developmental transcriptional regulatory network. Recent systems-level investigations of developmental transcriptional regulatory mechanisms have shown that the specification and differentiation programs of various cell types are encoded in enhancers that integrate and interconnect regulatory TFs to form an interactive network. These processes are orchestrated in pluripotent cells through the coordinated expression of the appropriate TFs and signaling proteins (that is, upstream components of the regulatory network) to generate a permissive cell state through which further differentiation can proceed through the coordinated expression of downstream effector genes (that is, downstream components of the regulatory network that mediate various aspects of terminal differentiation).

Homeodomain (HD) proteins are a large family of TFs that play a central role in establishing regional as well as tissue and single cellular fates [3]. A single HD TF is able to regulate different sets of downstream target genes depending on the developmental time and cell or tissue type in which it is expressed. Indeed, spatiotemporal context strongly influences whether particular target genes are activated or repressed by a given HD TF [3,4]. Recently, we have defined the complete spectrum of DNA sequences that are recognized by a large set of *Drosophila* HDs using protein binding microarrays (PBMs) [5]. This high-resolution analysis of HD DNA binding specificities revealed that numerous members of this TF structural class primarily recognize similar motifs (the canonical TAAT core sequence), but that individual HD TFs also preferentially bind related but unique sequences that are not recognized by other HDs [5,6,7]. In fact, we recently showed that different members of this class of TFs potentially determine the unique genetic programs of single cells through the selective recognition of particular DNA sequences that are preferred by one but not other HD proteins [5]. On the other hand, some HD subclasses, including the sine oculis homeobox (Six) subclass and certain members of the NK-2 HD subclass (Tinman (Tin) and Bagpipe (Bap)), recognize sequences which differ substantially from the canonical TAAT core sequence [5]. Aside from a few examples, the regulatory role of these DNA binding preferences have been largely uncharacterized [5,8]. Here we utilized the entirety of HD DNA binding specificities previously determined by PBMs to interrogate the individual contributions of different HD subclasses in regulating the activity of mesodermal enhancers that control the expression of both upstream and downstream components of mesodermal cell regulatory networks.

The specification and differentiation of the mesoderm in *Drosophila* leads to the formation of numerous distinct tissues including the heart and the somatic and visceral musculature [9]. Each tissue is composed of a diverse array of unique cell types. This has been most clearly shown with the larval somatic muscles, which are morphologically unique multinucleated myotubes [10]. Myotube identity originates in a population of mononucleated myoblasts termed founder cells (FCs) due to the combinatorial activities of muscle identity genes, many of which encode HD proteins [1,11]. A similar organ of extensive cellular diversity is the *Drosophila* heart, which is composed of two main cell types, the contractile cardial cells (CCs) and the surrounding pericardial cells (PCs). In fact, the majority of the PCs and CCs can be subdivided into individually unique cell types based on their specific TF gene expression patterns and the associated loss-of-function phenotypes of these TFs [8].

A subfamily of identity genes encoding HD TFs (referred to herein as identity homeodomains or I-HDs) has been shown to control the unique gene expression programs of individual mesodermal tissues and cells [5,12,13]. These genes belong to a diverse set of HD subclasses, including paired HD (Paired-type homeobox 1 (Ptx1)), Six (Six4), Iroquois (Caupolican (Caup)), NK-1 (Slouch (Slou), Ladybird late (Lbl), Lateral muscles scarcer (Lms)) and NK-2 (Tin, Bap) subclasses of HD TFs, as well as others such as Even skipped (Eve) and Muscle Segment Homeobox (Msh) [10]. Interestingly, recent systems-level studies have shown that I-HDs regulate both the upstream (for example, signaling molecules and TFs) and downstream (terminal differentiation) components of their gene regulatory networks [5,13,14,15]. The cellular identity functions of the I–HD TFs are distinct from the segment identity activities of the *Drosophila* Hox factors, which are also expressed in the mesoderm and involved in muscle and heart development [16,17,18]. A systems level analysis of the genes regulated by Hox TFs in the mesoderm has not been undertaken, though investigations in other tissues suggest the regulation of both upstream and downstream effector genes by Hox proteins [4,19].

**Figure 1:**
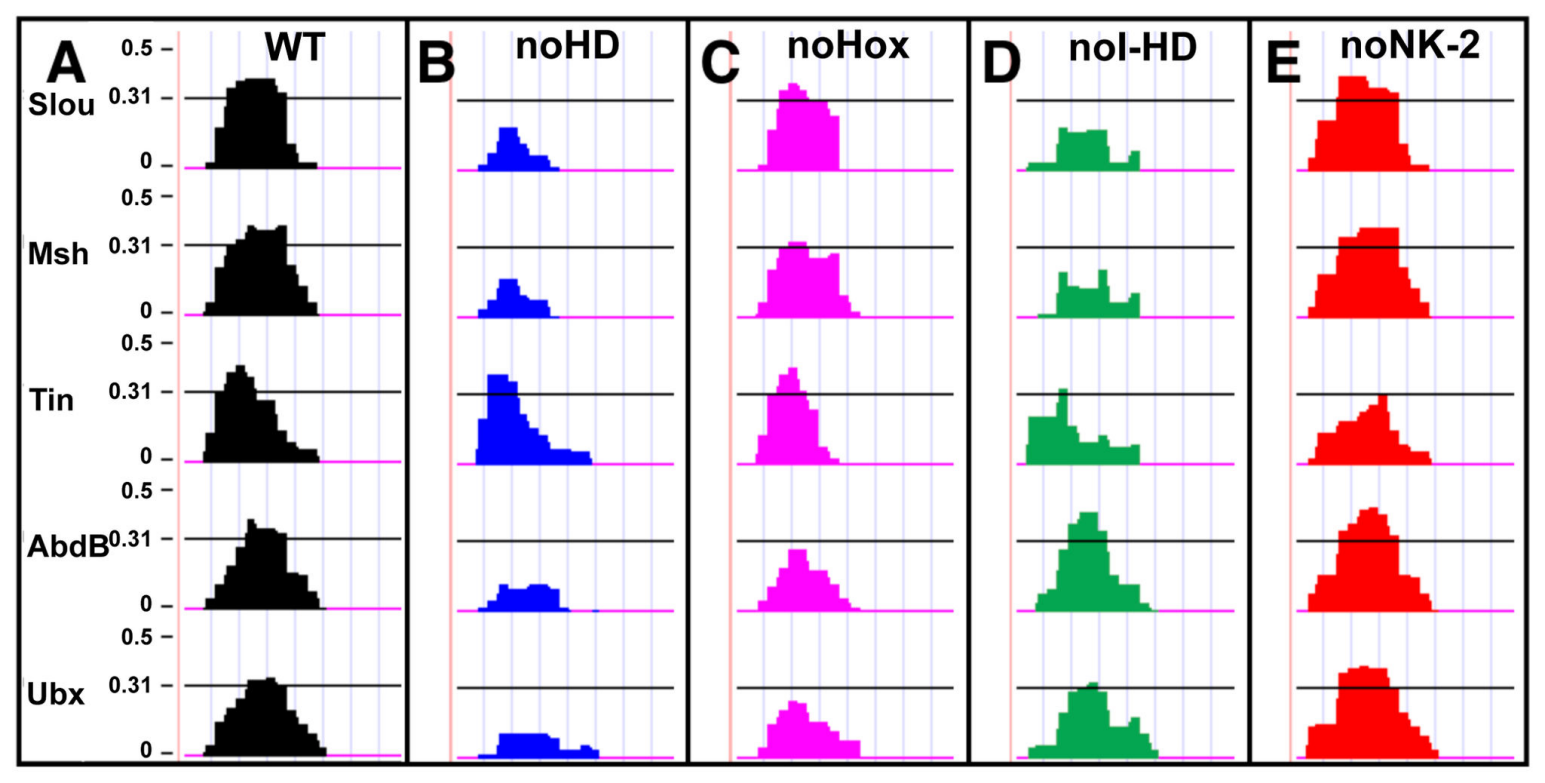
Targeted mutagenesis of different classes of HD binding sites in the *ap* muscle enhancer. E-score (y-axis) binding profiles of the indicated HD TFs within a particular 22 base pair segment of the entire wild-type (WT) *Ndg* enhancer (A) and versions in which all I-HD plus Hox (“noHD”, B), all Hox (“noHox”, C), or all I-HD (“noI-HD”, D) binding sites are mutated. The mutant in which all NK-2 binding sites are mutated are wild-type for these other HD TFs (“noNK-2”, E). The horizontal black line represents a threshold E-score of 0.31 below which binding is not considered significant, and was chosen as described in the Materials and Methods [5]. The effects of E-score binding profiles for additional HD TFs, as well as additional mutants investigated in the current study, and the entirety of the *Ndg* enhancer are shown in Figures S1-S4. See Materials and Methods for details of mutagenesis design and Table S2 for the actual nucleotide sequences that were investigated.

Despite exhibiting such discrete regulatory functions, many HD TFs have a restricted range of DNA binding specificities, which typically are centered on a canonical TAAT core [3]. The low information content of such DNA binding sites poses a challenge to understanding how HD TFs can mediate their precise developmental functions. In fact, using more recently published data [5], a systematic examination of the HD binding profile of the *ap* muscle enhancer, which was previously shown to be regulated by the Hox TF Antennapedia (Antp) through five putative Hox binding sites [16], reveals that these sites can also be recognized by Slou, Msh, Lbl, Eve, Ptx1, Caup, in addition to the the Hox factors Abdominal B (AbdB) and Ultrabithorax (Ubx) (see Figures 1 and S1-S4). Since many of these HD proteins are required for normal development of the *ap*-expressing muscles [16,20,21], it is therefore difficult to confidently and accurately assign a particular HD TF to the transcriptional response of the *ap* gene based on prior mutagenesis studies of individual HD DNA sequence motifs [16]. This problem necessitates that the question of which HDs actually provide regulatory input to enhancers be revisited by employing novel binding data that have only recently become available [5].

Thus, to evaluate the biological significance of different HD protein classes to the transcriptional regulation of different mesodermal genes, we took advantage of the comprehensive nature of recently published *in vitro* DNA binding specificity data to manipulate the ability of each subclass of HD to recognize mesodermal enhancers. Our results show that multiple subclasses of HD binding sequences, which represent binding sequences for Hox, I–HD, Six and NK-2 subclasses are independently required for the appropriate spatiotemporal activity of multiple mesodermal enhancers regulating both upstream regulatory and more downstream target genes. In total, these results demonstrate a requirement for the transcriptional integration of input from multiple HD subclasses in specifying cellular identities.

## Materials and Methods

### Fly Stocks

*Drosophila* stocks containing the following transgenes and mutant alleles were used: attP2 and nos-phiC31intNLS (gift of N. Perrimon, Harvard University, USA), *tin* ^346^ (gift of M. Frasch, University of Enlargen, Germany), *lbl-lacZ*, *mib2-lacZ*, and *Ndg-lacZ* [22].

### Cloning, Expression and Protein Binding Microarray Analysis of Caup

The DNA binding domain of Caup comprising residues 214-303 was cloned into a Gateway-compatible vector, and proteins were produced by in vitro transcription and translation (IVT PURExpress, New England Biolabs, Ipswich, MA, USA). PBM assays were performed as previously described [5,6,23] with 100 nM of Caup DBD applied to custom-designed microarrays (Agilent Technologies, AMADID 015681).

### Computational identification and mutagenesis of HD binding sites

We previously used PBM enrichment scores (E-scores) to identify sequences with binding capacity for individual HD proteins [5]. Our method involves the construction of 9-mer scores from the minimum E-score of the constituent 8-mers, and use of an E-score cutoff of 0.31 to identify candidate binding sequences; these parameters optimally separate bound from unbound sequences in a set of published footprinting experiments on *Drosophila* HD proteins [24]. These criteria were then used to screen candidate mutant enhancer sequences for those that abrogate binding of HDs of a given class while simultaneously preserving the ability of other HD TFs to bind to a given enhancer. Such experiments were designed to minimize the number of nucleotide changes in each enhancer sequence (from 0.402% to 4.98% of total nucleotides were changed, with the average being 3%). In addition, care was taken to avoid generating de novo binding sites for novel classes of transcriptional regulators found in UniPROBE [25]. Detailed E-score information relevant to the wild-type and mutant sequences shown for the enhancers in Table S2 can be found in Busser et al. [5] and in Table S1 for Caup.

### Analysis of Transgenic Reporter Constructs and Embryo Staining

Enhancer regions were synthesized *in vitro* (Integrated DNA Technologies, Coralville, IA, USA) and then subcloned into pWattB-GFP [5] or pWattB-nLacZ [26]. Constructs were targeted to attP40 [27] with phiC31-mediated integration [28]. Whole embryo immunohistochemistry and fluorescent in situ hybridization followed standard protocols [5]. The following antibodies were used: rabbit anti-Mef2 (1:1000, gift of B. Patterson), mouse anti-ßgal (1:500, Promega, Madison, WI), chicken anti-GFP (1:2000, Abcam, Cambridge, MA), guinea pig anti-Kr (1:300, gift of D. Kosman) and mouse anti-Ladybrid early (Lbe) (1:2500, gift of K. Jagla; Lbe and Lbl are co-expressed in the same mesodermal cells).

## Results

### Determination of the binding preferences for Caup

We previously used protein binding microarrays (PBMs) to define the complete spectrum of DNA binding preferences of a large set of HDs that are expressed in the *Drosophila* embryonic mesoderm where they are known from genetic studies to have a variety of developmental functions [5]. However, these studies did not include the Iroqouis subclass HD Caup, which is known to play a critical role in myogenesis [20]. To determine the in vitro DNA binding preferences of Caup, we used PBMs containing replicates of all possible 8-mer DNA sequences and followed a standardized protocol [5,6,23]. We generated a position weight matrix (PWM) from the bound sequences to visualize the DNA binding preferences of Caup (Table S1). These results show that the in vitro binding preferences of Caup are very similar to both mouse homologs of this TF and a previous analysis of Caup using a bacterial one-hybrid system [6,7]. In addition, the PBM data revealed that Caup exhibited DNA binding preferences that are distinct from the majority of HDs, which primarily recognize sequences centered around TAAT (Table S1) [5].

### Identification and selective mutagenesis of I–HD, Hox, and NK-2 subclasses of HD binding sites in mesodermal enhancers

Our previous analysis of the DNA binding preferences of *Drosophila* HDs included members of the Hox family (Ubx, AbdB) which are important in the establishment of segmental identity [18], as well as paired, Six, and NK families which are involved in establishing the identities of individual cells (I-HDs) [10]. Further, we have now determined the binding preferences for an Iroquois subclass HD, which has recently been shown to be an I–HD TF [20]. These studies revealed extensive overlap in the binding specificities of numerous HDs centered around a TAAT core [5]. As both Hox and I–HD TFs are critical for mesodermal gene expression, this redundancy in binding complicates the ability to confidently assign the role of a binding sequence that is recognized by multiple TFs to a particular HD subclass when using *in vitro* mutagenesis and transgenic reporter assays to determine the functions of particular sites. In addition, the role of HD subclasses which recognize sequences which differ from the canonical TAAT core sequence (such as NK-2) have been largely unexplored in regulating broad mesodermal gene expression patterns.

**Figure 2:**
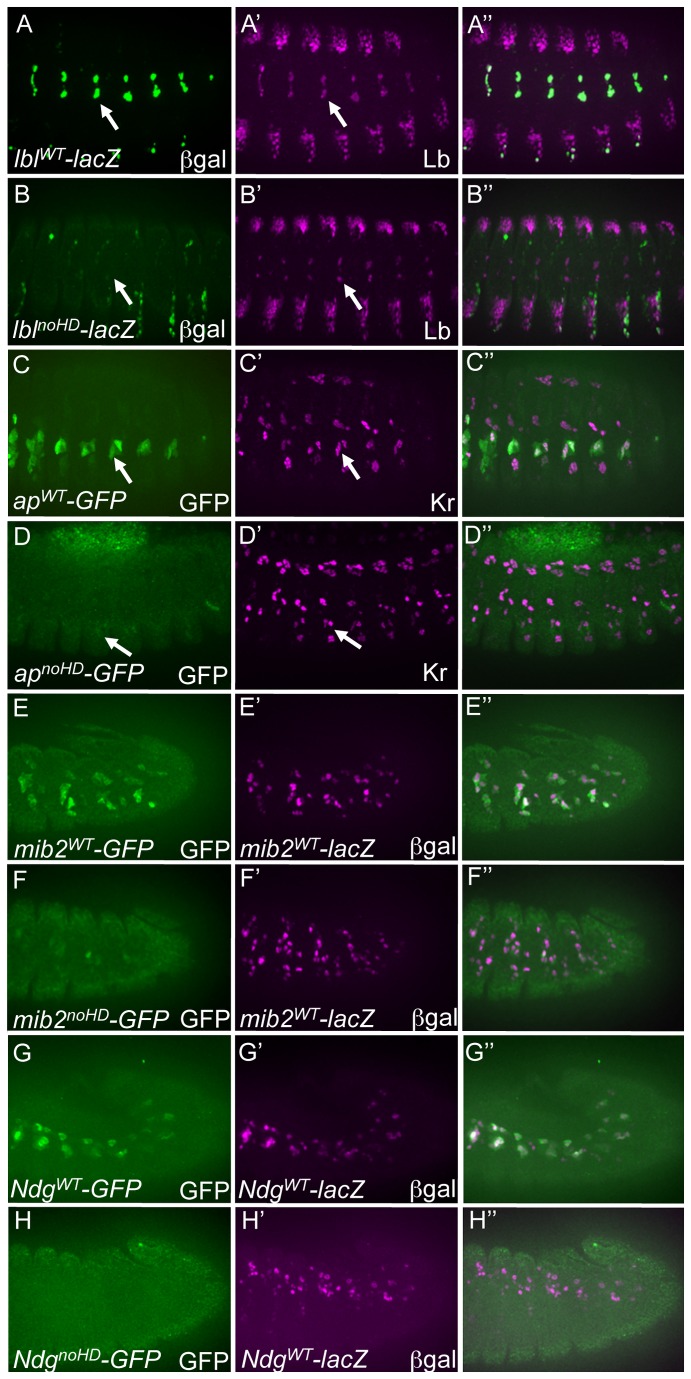
Functional requirements for HD binding sites in all tested mesodermal enhancers. (A) ßgal (green) driven by the wild-type (WT) *lbl* enhancer (*lbl^WT^-lacZ*) co-expresses with Lb protein (magenta) in the Lb-expressing SBM in stage 14 embryos. (B) Loss of ßgal reporter in the Lb-expressing SBM driven by a version of the *lbl* enhancer in which all I–HD plus Hox binding sites are selectively inactivated (*lbl^noHD^-lacZ*) in stage 14 embryos. (C) The GFP (green) reporter driven by the WT *ap* enhancer (*ap^WT^-GFP*) is active in stage 14 lateral transverse myotubes, two of which express Kr protein (magenta). (D) Loss of the GFP reporter in stage 14 lateral transverse myotubes by a version of the *ap* enhancer in which all I–HD plus Hox binding sites are inactivated (*ap^noHD^-lacZ*). (E) GFP (green) and ßgal (magenta) are co-expressed in stage 12 embryos when driven by the WT *mib2* enhancer (*mib2* ^*WT*^ *-GFP* and *mib2* ^*WT*^ *-lacZ*, respectively). (F) GFP (green) expression driven by a version of the *mib2* enhancer in which all I–HD plus Hox binding sites are mutated (*mib2^noHD^-GFP*) is significantly reduced compared to ßgal (magenta) driven by *mib2* ^*WT*^ *-lacZ* in stage 12 embryos. (G) GFP (green) and ßgal (magenta) are co-expressed when driven by the *Ndg* enhancer in stage 12 embryos (*Ndg* ^*WT*^ *-GFP* and *Ndg* ^*WT*^ *-lacZ*, respectively). The 1-2 non-co-expressing cells are due to ectopic reporter activity caused by the P-element insertion [22]. The ventral *Ndg* reporter-expressing cells are not present in the indicated focal plane. (H) Loss of GFP (green) driven by a version of the *Ndg* enhancer in which all I–HD plus Hox binding sites are mutated (*Ndg^noHD^-GFP*) as compared to ßgal (magneta) driven by the WT *Ndg* enhancer (*Ndg^WT^-lacZ*) in stage 12 embryos. The ventral *Ndg* reporter-expressing cells are not in this focal plane but do not express the GFP reporter (data not shown).

**Figure 3:**
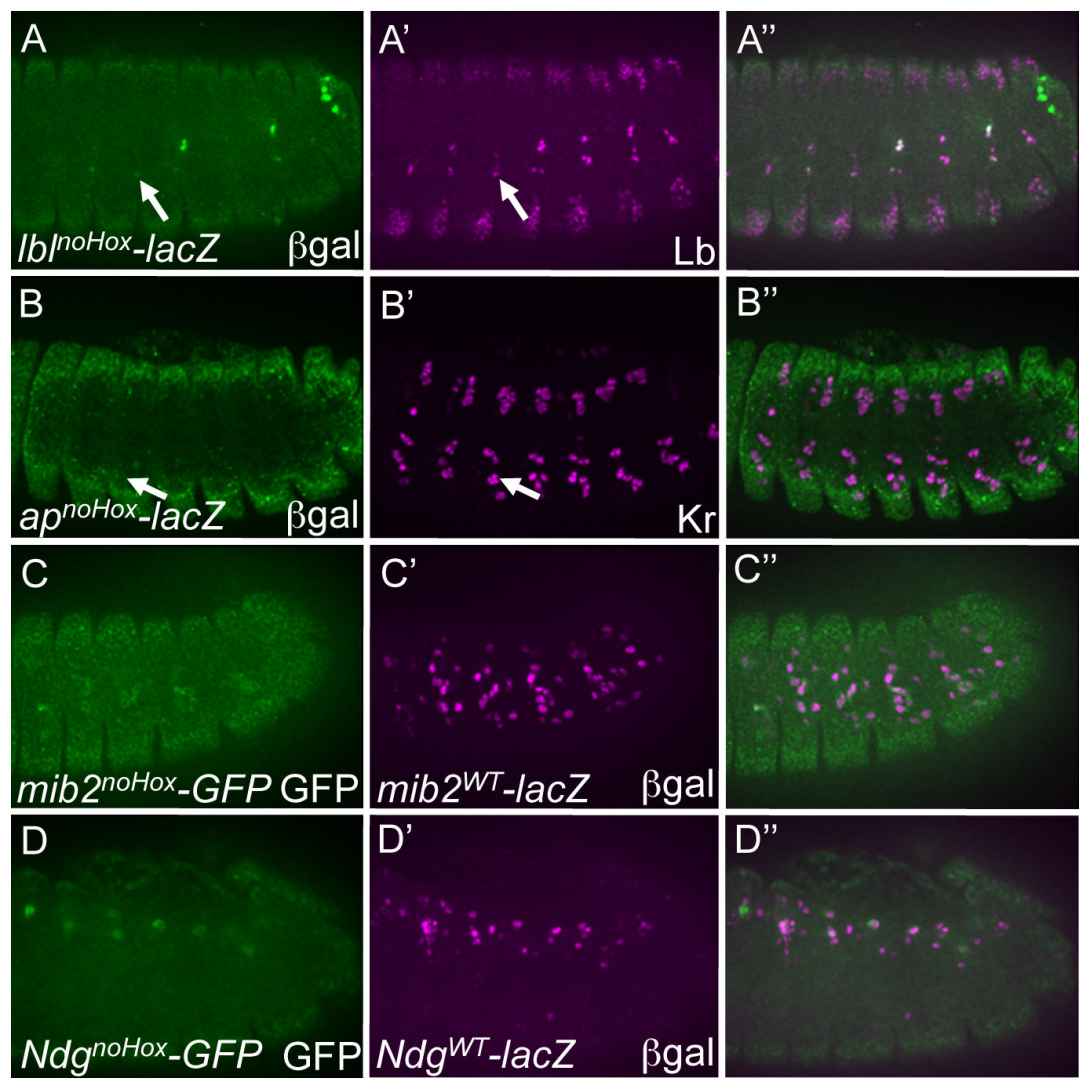
Hox binding sites are required for the full activities of all tested mesodermal enhancers. (A) Loss of ßgal reporter (green) in the Lb-expressing SBM (magneta) driven by a version of the *lbl* enhancer in which the Hox binding sites are mutated (*lbl^noHox^-lacZ*) in stage 14 embryos. Compare to the WT version of the *lbl* enhancer (*lbl^WT^-lacZ*) in Figure 2A. (B) Loss of ßgal reporter (green) in the *ap* enhancer in which the Hox binding sites are mutated (*ap^noHox^-lacZ*) in stage 14 embryos. Compare to the WT version of the *ap* enhancer (*ap^WT^-lacZ*) in Figure 2C. (C) Attenuation of GFP (green) driven by a version of the *mib2* enhancer in which Hox binding sites are inactivated (*mib2^noHox^-GFP*) as compared to ßgal (magneta) driven by a WT version of the *mib2* enhancer (*mib2^WT^-lacZ*) in stage 12 embryos. Compare to WT versions of both GFP and lacZ reporters in Figure 2E. (D) Attenuation of GFP (green) driven by a version of the *Ndg* enhancer in which Hox binding sites are inactivated (*Ndg^noHox^-GFP*) as compared to ßgal (magneta) driven by a WT version of the *Ndg* enhancer (*Ndg^WT^-lacZ*) in stage 12 embryos. The ventral *Ndg* reporter-expressing cells are not in the indicated focal plane but do not express the GFP reporter (data not shown). Compare to WT versions of both GFP and lacZ reporters in Figure 2G.

To test the separate and distinct contributions of Hox, I–HD and NK-2 TF binding sites to mesodermal gene regulation, we used the complete spectrum of DNA binding preferences compiled from PBM data to identify and to selectively generate by *in vitro* mutagenesis nonbinding versions of predicted Hox, I–HD and NK-2 recognition sequences in a number of well-characterized mesodermal enhancers, while simultaneously preserving to the greatest extent possible the pattern of binding sequences for other classes of HD TFs (see Figures 1 and S1-S4) [5,26]. In addition, experiments were designed to minimize the number of nucleotide changes in each enhancer sequence, which varied from 0.402% to 4.98% of total nucleotides changed (average = 3%), which is comparable to the number of nucleotide changes in enhancer sequences in previous investigations of Hox function, which varied from 0.66% to 5.4% of total nucleotides changed (average = 3.2%) [16,29,30,31,32,33,34]. A representative example is shown in Figure 1 in which the PBM-derived enrichment scores (E-scores) of different HD classes are mapped along a segment of the *Ndg* enhancer, with the horizontal black line representing a threshold binding E-score > 0.31, which we previously showed optimally separated bound from unbound sequences (see Materials and Methods) [5]. In this example, the wild-type (WT) stretch of the enhancer is bound by both I–HD (represented by Slou and Msh) and Hox TFs (represented by AbdB and Ubx). In addition, a series of enhancers were generated which: (1) lacked the ability to be recognized by either Hox (these include Ubx and AbdB) or I–HD (these include Slou, Msh, Lbl, Eve, Ptx1, Six4, and Caup) TFs, but could still be recognized by NK-2 TFs (these include Tin and Bap); this category of mutant enhancers is referred to as “noHD” (Figure 1); (2) lacked the ability to be recognized by Hox TFs but could still be bound by I–HD TFs and NK-2 TFs (referred to as “noHox”; (Figure 1); (3) lacked the ability to be recognized by I–HD TFs but could be still recognized by Hox TFs and NK-2 TFs (referred to as “noI-HD”; (Figure 1); and (4) lacked the ability to be recognized by NK-2 HD TFs but could still be recognized by Hox and I–HD TFs (referred to as “noNK-2”; (Figure 1).

We focused our studies on previously characterized mesodermal enhancers with broad expression domains, known from genetic studies to be regulated by I–HD, Hox and NK-2 TFs, and associated with genes from both upstream and downstream components of the mesodermal gene regulatory network. Thus, we performed our analyses on the enhancers for *ap* and *lbl*, which represent more upstream components of the myoblast regulatory network since they both encode developmentally important TFs, plus *mib2* (an E3 ubiquitin ligase) and *Ndg* (a basement membrane protein), which represent more downstream components of the mesodermal gene regulatory network [1,16,22]. These enhancers are active in a diverse array of mesodermal cells, with the *lbl* reporter active in a FC which corresponds to the segment border muscle (SBM) and two adult muscle precursors per hemisegment (see Figure 2A) [5,22], while the *ap* muscle enhancer is active in a nearby lateral cluster of FCs which correspond to lateral transverse muscles 1-4 (see Figure 2C) [16]. The *mib2* (see Figure 2E) and *Ndg* (see Figure 2G) enhancers encompass a broader array of embryonic cells, with both *mib2* and *Ndg* active in subsets of FCs and the gut musculature, as well as two different cardial cells per hemisegment, while the *Ndg* enhancer is also active in two pericardial cells and two cells of the central nervous system (which are not of mesodermal origin) per hemisegment [22,35]. The *ap*, *mib2* and *Ndg* enhancers are relatively small (on average, 690 bp, Table S2), while the initially characterized *lbl* enhancer was much larger (1350 bp) [22]. In order to define a shorter enhancer for this latter gene, we tested an evolutionarily-conserved subfragment (854 bp) which we found directed reporter activity in the same three mesodermal cells (see Figure 2A). Finally, all of these genes are known targets of the I–HD TFs Slou and Msh [5] as well as the NK-2 TF Tin [15,36], and both *Ndg* and *ap* are regulated by Hox TFs [4,16]. Moreover, the expression patterns of these genes suggest further regulation by additional I–HD and Hox TFs [18,20,37,38,39]. To compare activities of the different enhancer constructs, we either stained with appropriate markers to monitor the reporter activity, or crossed *Drosophila* strains with wild-type or mutant reporters to each other [5,26].

### Mesodermal enhancer activity requires sequences capable of recognition by either Hox or I–HD TFs

To test the role of both I–HD and Hox input to mesodermal enhancers, we first examined the effects of mutating all binding sites with the ability to be recognized by either Hox or I–HD TFs while preserving their ability to be recognized by NK-2 TFs (see Figure 1B; so-called “noHD” constructs). Mutagenesis of all I–HD plus Hox binding sequences significantly affected the activity of all reporters (Figure 2). In the versions of the *lbl* (*lbl* ^noHD^, Figure 2B) *ap* (*ap* ^noHD^, Figure 2D), and *Ndg* (*Ndg* ^*noHD*^, Figure 2H) enhancers unable to be recognized by I–HD plus Hox proteins, there is a complete abrogation of reporter activity as compared to wild-type versions of those enhancers (*lbl* ^*WT*^, Figure 2A; *ap* ^*WT*^, Figure 2C; *Ndg* ^*WT*^, Figure 2G), while only a few isolated cells retain reporter activity in the *mib2* enhancer having mutant I–HD and Hox binding sites (*mib2* ^*noHD*^, Figure 2F) as compared to the wild-type enhancer (*mib2* ^*WT*^, Figure 2E). In total, these results support the hypothesis that transcriptional input from both Hox and/or I–HD TFs are critical for generating appropriate patterns of mesodermal gene expression, independent of any activity provided by the separate class of NK-2 HDs with their distinct DNA binding preferences.

### Hox binding sites alone are necessary for mesodermal enhancer activity

As there is extensive overlap amongst I–HD and Hox TFs (Figures 1 and S1-S4), the preceding results do not discriminate between the role of Hox and I–HD TFs in mediating mesodermal gene expression. To assess the potentially independent effects of these two HD subclasses in regulating mesodermal enhancers, we first tested the role of Hox TFs by selectively mutating predicted Hox TF binding while preserving the ability of I–HD and NK-2 TFs to recognize the four mesodermal enhancers (so-called “noHox” constructs; Figure 1C). These results show that the selective mutagenesis of Hox binding sites alone largely inactivates all mesodermal reporters (Figure 3). For example, the *lbl* ^noHox^ (Figure 3A) and *ap* ^noHox^ (Figure 3B) are almost completely inactive as compared to wild-type versions of those enhancers (*lbl* ^*WT*^, Figure 2A and *ap* ^*WT*^, Figure 2C), with the exception of a few isolated Lbl-positive cells that retain reporter activity in *lbl* ^noHox^ embryos (Figure 3A). Likewise, the *mib2* ^*noHox*^ (Figure 3C) and *Ndg* ^*noHox*^ (Figure 3D) GFP reporters are only active in a few cells as compared to the corresponding wild-type lacZ versions (*mib2* ^*WT*^, Figure 3C’ and *Ndg* ^*WT*^, Figure 3D’). In addition, the activity of the *mib2* ^*noHox*^ and *Ndg* ^*noHox*^ enhancers were variable in each hemisegment and between different transgenic embryos of the same genotype, which further suggests that enhancer activity is spatially imprecise and stochastic at the single cell level in the absence of Hox input. In total, these results suggest that the transcriptional input from Hox TFs is integrated at mesodermal enhancers for multiple genes representing distinct layers of the mesodermal gene regulatory network, independent of input from other HD classes that bind to and act on these same enhancers.

### I–HD binding sites alone are necessary for mesodermal enhancer activity

**Figure 4:**
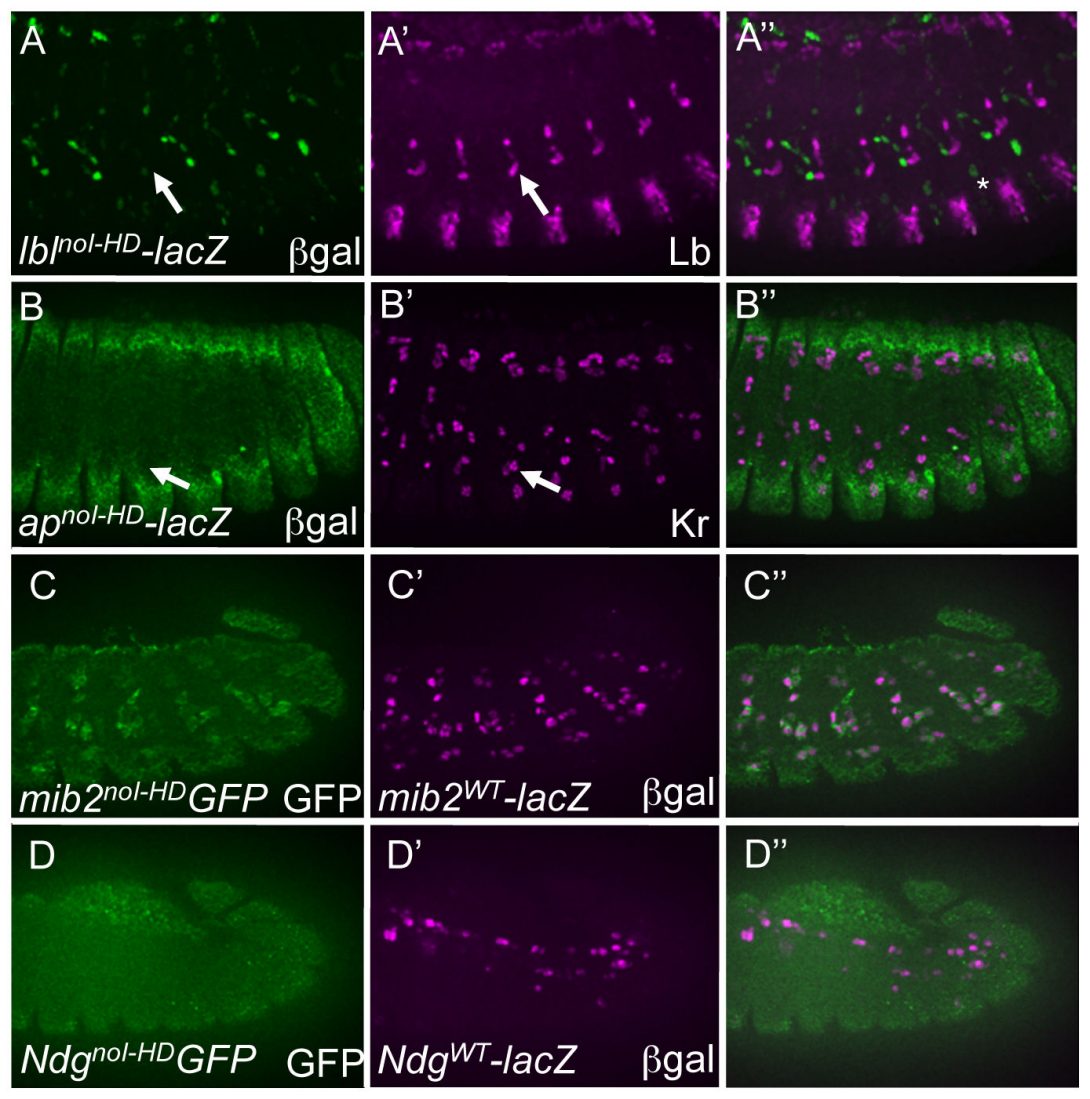
I–HD binding sites are required for the full activities of all tested mesodermal enhancers. (A) Loss of ßgal reporter (green) in the Lb-expressing SBM (magneta) driven by a version of the *lbl* enhancer in which the I–HD binding sites are inactivated (*lbl^noI-HD^-lacZ*) in stage 14 embryos. Compare to the WT version of the *lbl* enhancer (*lbl^WT^-lacZ*) in Figure 2A. Asterix indicate ßgal-expressing myotube VT1. (B) Loss of ßgal reporter (green) in the *ap* enhancer in which the I–HD binding sites are mutated (*ap^noI-HD^-lacZ*) in stage 14 embryos. Compare to the WT version of the *ap* enhancer (*ap^WT^-lacZ*) in Figure 2C. (C) Attenuation of GFP (green) driven by a version of the *mib2* enhancer in which FCI-HD binding sites are inactivated (*mib2^noI-HD^-GFP*) as compared to ßgal (magneta) driven by a WT version of the *mib2* enhancer (*mib2^WT^-lacZ*) in stage 12 embryos. Compare to WT versions of both GFP and lacZ reporters in Figure 2E. (D) Loss of GFP (green) driven by a version of the *Ndg* enhancer in which I–HD binding sites are inactivated (*Ndg^noI-HD^-GFP*) as compared to ßgal (magneta) driven by a WT version of the *Ndg* enhancer (*Ndg^WT^-lacZ*) in stage 12 embryos. The ventral *Ndg* reporter-expressing cells are not present in the indicated focal plane but do not express the GFP reporter (data not shown). Compare to WT versions of both GFP and lacZ reporters in Figure 2G.

We next investigated the role of I–HD TFs in regulating mesodermal gene expression by selectively mutating predicted I–HD TF binding sequences in the mesodermal enhancers, while preserving predicted Hox TF binding sequences (so-called “noI-HD” constructs; Figure 1D). The *in vivo* functions of these mutant enhancers show that I–HD binding sites are also separately required for the appropriate mesodermal activity of all investigated enhancers. For example, I–HD binding sites are absolutely required for the reporter activity of all cells in both the *ap* (*ap* ^noI-HD^, Figure 4B) and *Ndg* (*Ndg* ^*noI-HD*^, Figure 4D) enhancers as compared to wild-type versions of those reporters (*ap* ^*WT*^, Figure 2C and *Ndg* ^*WT*^, Figure 4D’). In the absence of I–HD binding sites, the activity of the *mib2* (*mib2* ^*noI-HD*^, Figure 3C) enhancer was not completely extinguished but rather was significantly attenuated in all cells as compared to the wild-type reporter (Figure 3C’, *mib2* ^*WT*^). The *lbl* enhancer (*lbl* ^noI-HD^, Figure 4A) presents an interesting example, as reporter activity is completely lost from the normal expressing cells (the Lb-expressing SBM and two AMPs), but the mutant I–HD reporter now becomes weakly active in another myotube (ventral transverse muscle 1, VT1) which normally expresses the I–HD Slou [5]. We previously showed the necessity of a binding site which was preferentially recognized by Slou in the *lbl* enhancer was required for the normal restriction of *lbl* reporter activity to the SBM [5]. Thus, this new result shows that the *lbl* enhancer requires I–HD binding sites for the normal activation and restriction of the enhancer to three mesodermal cells. In total, these results confirm a direct regulatory role for I–HD TFs in the regulation of multiple genes representing both upstream and downstream components of mesodermal gene regulatory networks, independent of Hox input to these same enhancers.

### Six binding sequences are required for enhancer activity associated with the expression of some but not all mesodermal genes

Previous studies have shown a requirement for the *Drosophila* homolog of the myotonic dystrophy type 1-associated HD Six5 (in *Drosophila* , D-Six4 is the closest homologue to Six5) in the proper specification of lateral and ventral mesodermal structures, which include the somatic musculature [40]. We previously showed that many members of the HD family largely recognize similar AT-rich sequences [5]. However, the binding profile of Six4 deviates substantially from this canonical TAAT core sequence [5]. As the preceding analyses of I–HD function included Six binding sequences, we now asked specifically whether the Six class of HDs is independently required for the proper activity of mesodermal enhancers. To do so, we mutated predicted Six4 binding sequences in otherwise wild-type versions of the same four mesodermal enhancers (see Table S2 for the sequences of Six4 binding sites and details of their mutagenesis in our test system).

**Figure 5:**
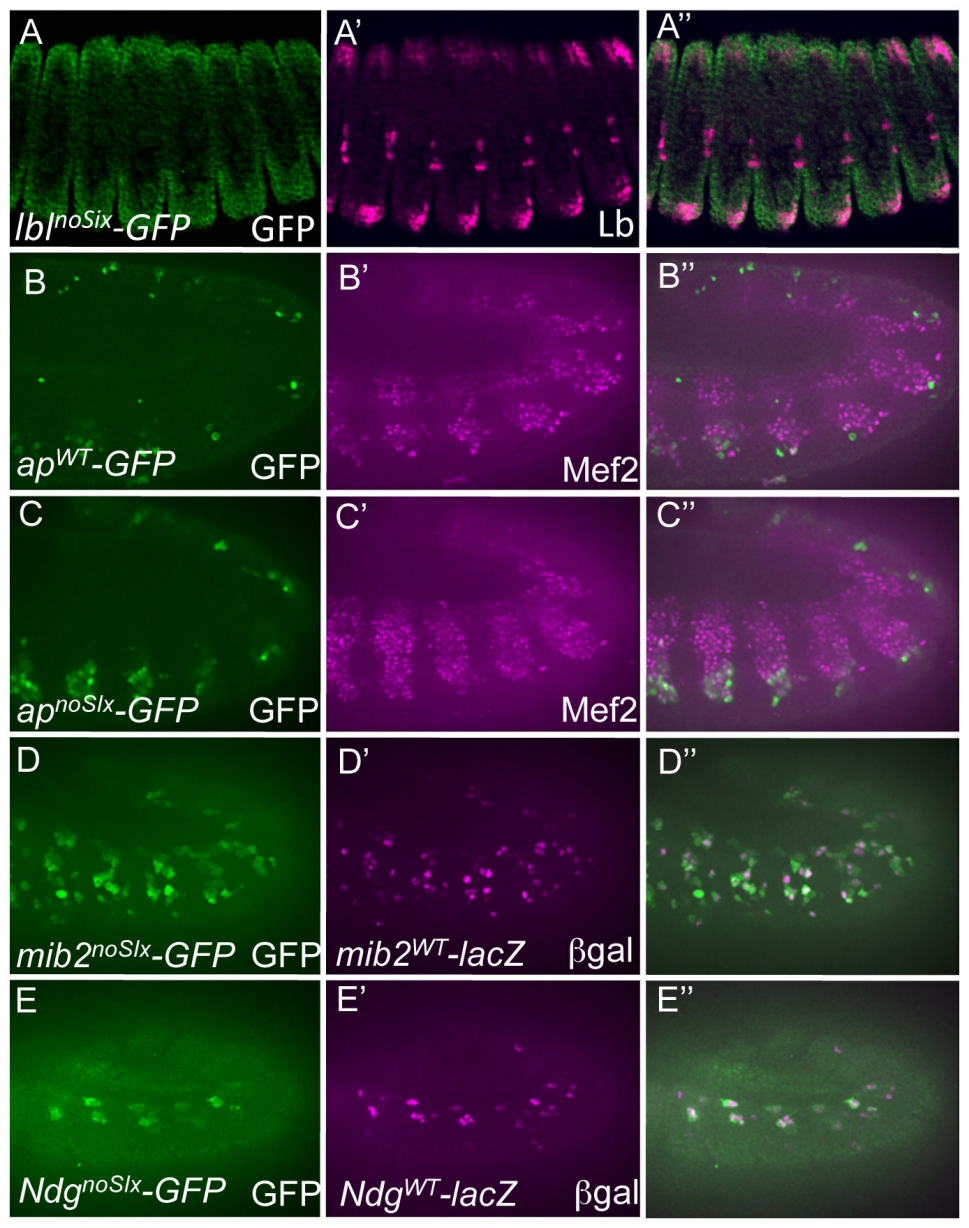
Requirements of Six binding sites for the activities of some but not all tested mesodermal enhancers. (A) Loss of GFP (green) reporter expression in the Lb-expressing SBM (magneta) driven by a version of the *lbl* enhancer in which the Six4 binding sites are inactivated (*lbl^noSix^-GFP*) in stage 14 embryos. Compare to the WT version of the *lbl* enhancer (*lbl^WT^-lacZ*) in Figure 2A. (B) The GFP (green) reporter driven by the WT *ap* enhancer (*ap^WT^-GFP*) is active in a small subset of lateral Mef2-positive FCs (magenta) in stage 12 embryos. (C) De-repression of the GFP reporter (green) into additional Mef2-positive (magenta) mesodermal cells in a version of the *ap* enhancer in which the Six4 binding sites are mutated (*ap^noSix^-GFP*) in stage 12 embryos. (D) The GFP (green) reporter driven by a version of the *mib2* enhancer in which Six4 binding sites are inactivated (*mib2^noSix^-GFP*) co-expresses with ßgal (magneta) driven by a WT version of the *mib2* enhancer (*mib2^WT^-lacZ*) in stage 12 embryos. Compare to WT versions of both GFP and lacZ reporters in Figure 2E. (E) The GFP (green) reporter driven by a version of the *Ndg* enhancer in which Six4 binding sites are mutated (*Ndg^noSix^-GFP*) also co-expressed with ßgal (magneta) driven by a WT version of the *Ndg* enhancer (*Ndg^WT^-lacZ*) in stage 12 embryos. The ventral *Ndg* reporter-expressing cells are not present in the indicated focal plane but do not express the GFP reporter (data not shown). Compare to WT versions of both GFP and lacZ reporters in Figure 2G.

It was previously shown that the Six4 gene is required for the proper development of the SBM and expression of the Lb gene [40]. We now show that the effects of Six4 are mediated by the *lbl* FC enhancer, since mutagenesis of the two Six4 binding sites in this enhancer completely extinguished its activity (*lbl* ^noSix^, Figure 5A) as compared to the wild-type reporter (*lbl* ^*WT*^, Figure 2A). On the other hand, the Six4 binding sites in the *ap* enhancer were not required for activation of the reporter, but rather restricted the reporter to the correct mesodermal cells, as mutagenesis of the Six4 binding sites in the *ap* enhancer (*ap* ^noSix^, Figure 5C) induced de-repression of the reporter into additional mesodermal cells as compared to the wild-type reporter (*ap* ^*WT*^, Figure 5B). Interestingly, Six binding sequences were not required in either of the enhancers for the more downstream components (*mib2* and *Ndg*), in spite of their being active in domains of the mesoderm regulated by Six4 (Figure 5). For example, we show that mutagenesis of the Six binding sites in the *mib2* (*mib2* ^*noSix*^, Figure 5D) and *Ndg* (*Ndg* ^*noSix*^, Figure 5E) enhancers were entirely comparable to their wild-type counterparts (*mib2* ^*WT*^, Figure 5D’ and *Ndg* ^*WT*^, Figure 5E’). In total, these results suggest that Six4 is required for the correct expression of some but not all components of mesodermal gene regulatory networks.

### Requirements for NK-2 HD binding sequences in mesodermal enhancers

Tin belongs to the NK-2 subclass of HDs and is required for the proper specification of dorsal mesodermal derivatives including the heart, gut musculature and dorsal somatic muscles [6,41,42]. Interestingly, Tin is also required for the proper development of numerous ventral and lateral somatic muscles, even though Tin expression is entirely restricted to the dorsal mesoderm at the time when these muscle FCs are specified [41]. In fact, recent studies confirmed that Tin is bound *in vivo* to the *mib2, Ndg* and *ap* enhancers [43]. For this reason, and also the fact that the binding specificity of Tin is highly distinct from that of the other I–HD TFs, we investigated the specific contribution of this subclass of HDs to mesodermal gene expression [5,41]. For these analyses, we included another NK-2 HD Bap, which has a highly similar binding profile as Tin, and is required for the proper specification of the gut musculature. To investigate the contribution of Tin/Bap to mesodermal gene expression, we utilized site-directed mutagenesis of only NK-2 binding sites while simultaneously preserving the ability of I–HD and Hox TFs to recognize these enhancers (so-called “noNK-2” constructs; Figure 1D).

**Figure 6:**
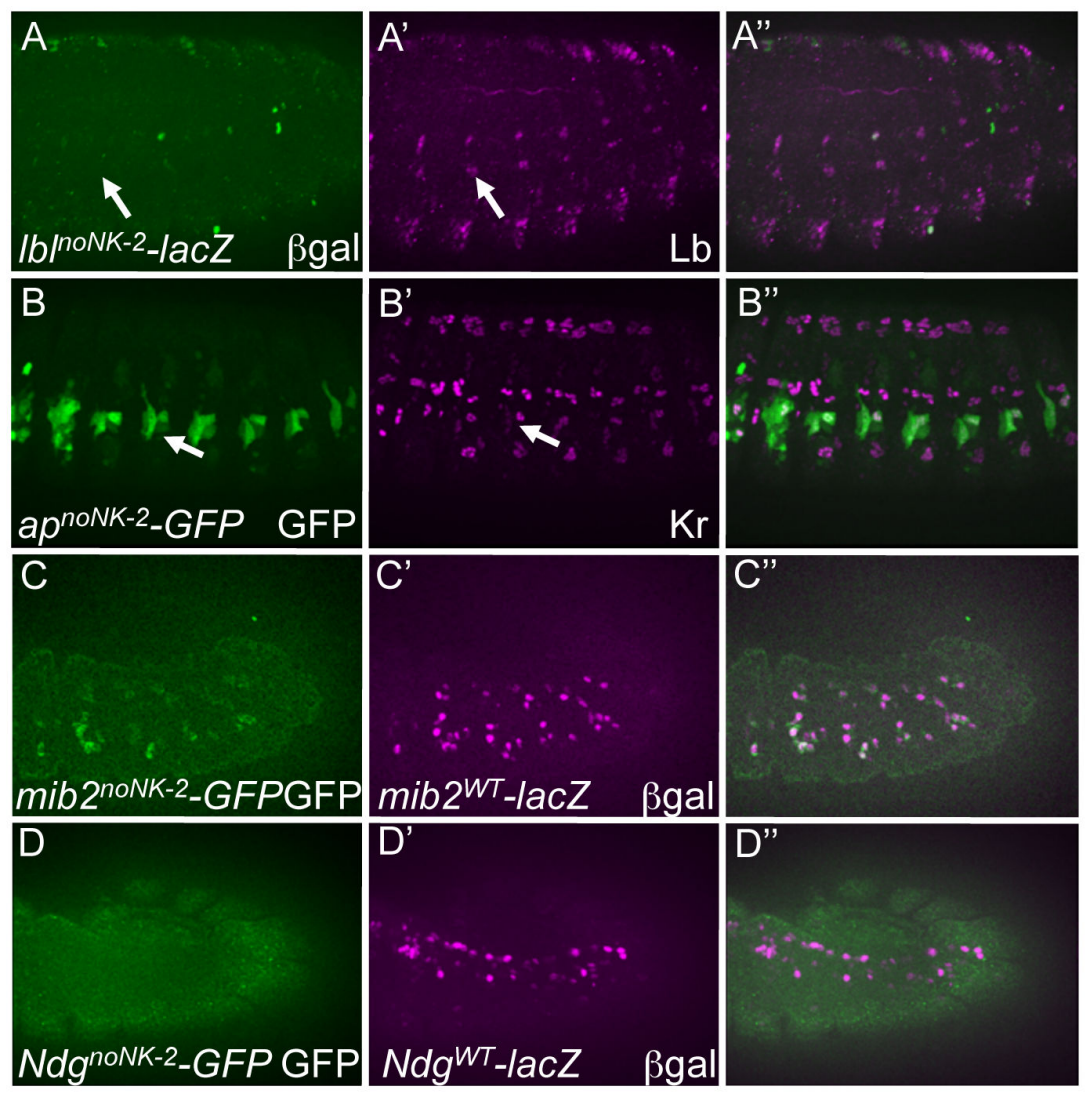
Requirements for NK-2 binding sites for the full activities of multiple tested mesodermal enhancers. (A) Loss of ßgal reporter (green) in the Lb-expressing SBM (magneta) driven by a version of the *lbl* enhancer in which the Tin binding sites are inactivated (*lbl^noNK-2^-lacZ*) in stage 14 embryos. Compare to the WT version of the *lbl* enhancer (*lbl^WT^-lacZ*) in Figure 2A. (B) Normal GFP reporter (green) activity in the *ap* enhancer in which the Tin binding sites are mutated (*ap^noNK-2^-GFP*) in stage 14 embryos. Compare to the WT version of the *ap* enhancer (*ap^WT^-GFP*) in Figure 2C. (C) Attenuation of GFP (green) driven by a version of the *mib2* enhancer in which Tin binding sites are inactivated (*mib2^noNK-2^-GFP*) as compared to ßgal (magneta) driven by a WT version of the *mib2* enhancer (*mib2^WT^-lacZ*) in stage 12 embryos. Compare to WT versions of both GFP and lacZ reporters in Figure 2E. (D) Loss of GFP (green) driven by a version of the *Ndg* enhancer in which Tin binding sites are mutated (*Ndg^noNK-2^-GFP*) as compared to ßgal (magneta) driven by a WT version of the *Ndg* enhancer (*Ndg^WT^-lacZ*) in stage 12 embryos. The ventral *Ndg* reporter-expressing cells are not in this focal plane but do not express the GFP reporter (data not shown). Compare to WT versions of both GFP and lacZ reporters in Figure 2G.

The corresponding functional assays revealed that NK-2 binding is required for wild-type activity of the *mib2*, *Ndg* and *lbl* but not the *ap* enhancers (Figure 6). Site-directed mutagenesis of the NK-2 binding sites in the *lbl* enhancer (*lbl* ^noNK-2^, Figure 6A) largely abrogated reporter activity in the Lb-expressing SBM, with only minor levels detected in a few hemisegments as compared to the wild-type enhancer (*lbl* ^*WT*^, Figure 2A). Similarly, inactivation of NK-2 binding sites in the *Ndg* enhancer (*Ndg* ^*noNK-2*^, Figure 6D) completely inactivated the reporter in all domains as compared to the WT enhancer (*Ndg* ^*WT*^, Figure 6D’). In addition, reporter activity in *mib2* ^*noNK-2*^ (Figure 6C) mutant embryos was highly abnormal, with only a minority of cells expressing wild-type levels of the reporter (*mib2* ^*WT*^, Figure 6C’). On the other hand, since there are no identifiable Tin binding sites and only weak Bap sites in the *ap* enhancer that is active in somatic but not in visceral myoblasts, it is not surprising that there was no effect on enhancer activity of mutagenizing the latter sites (*ap* ^noNK-2^, Figure 6B) as compared to the wild-type version of the enhancer (*ap* ^*WT*^, Figure 2C). Interestingly, as Tin protein is known to be bound to the *ap* enhancer [43], these results suggest that such binding is not mediated by direct TF-DNA interactions. However, recent studies have shown that protein cofactors can alter TF DNA-binding specificity, raising the possibility that cofactors affect Tin-binding specificity in the case of the *ap* enhancer [44,45]. Since novel Tin specificities generated in this manner would not have been included in our analysis of NK-2 binding sites in the *ap* enhancer, it remains possible that such binding sites are indeed required for activity of this enhancer. In total, these results document a critical role for direct NK-2 binding to multiple mesodermal enhancers to orchestrate their appropriate activity, independent of the dorsoventral location of the myoblasts in which the enhancer of interest is normally active.

## Discussion

We have examined the molecular basis of the ability of multiple classes of HD TFs to independently regulate specific gene expression programs despite the tendency of many of these proteins to bind to similar DNA sites in vitro. A comprehensive catalog of DNA binding specificities [5] was used to selectively manipulate the ability of an individual enhancer to be recognized by I–HD or Hox TFs. These studies unambiguously demonstrate that both I–HD and Hox TFs provide independent input to multiple mesodermal enhancers, a conclusion that was not possible to make from previous studies in which unique and overlapping HD binding site specificities were not taken into account in the design of appropriate functional experiments, thus failing to distinguish between HD classes based on types of DNA recognition sequences [16,29,30,31,32,33,34].

Our studies revealed that the effects on reporter activity in the absence of Hox input were not restricted to the individual segments in which AbdB and Ubx are expressed. This suggests that the analysis of Hox binding sequences in these studies included other specificities such as AbdA and Antp, as the *mib2*, *Ndg*, *ap* and *lbl* enhancers are active in these segments. In addition, as the absence of Hox binding sites caused a loss of reporter activity, this suggests that Hox TFs are acting as activators of segment-specific gene expression programs. Due to the absence of ectopic enhancer activity, it would appear that the Hox TFs are not acting as repressors in the mesoderm, at least not for the genes whose mesodermal enhancers we examined. However, it is important to consider that the present work could not analyze the contribution of an individual Hox TF due to the substantial overlap in their DNA binding specificities [5,6,7], which would negate the confounding influence of other Hox HDs in mediating segment-specific enhancer activities.

The analysis of I–HD contribution to mesodermal gene activity included Iroqouis (Caup), NK-1 (Slou), paired (Ptx1), Six (Six4) and other HDs (Msh and Eve), each of which are expressed in different subsets of mesodermal cells [10]. The predominant phenotype in the absence of I–HD binding was loss of reporter activity, which suggests that I-HDs are activating these enhancers in the individual mesodermal cell types where the particular I–HD TF is expressed. However, the general absence of ectopic enhancer activity should be cautiously interpreted for the same reason as that described for Hox TFs (that is, the inability to investigate individual I-HDs owing to overlapping DNA binding specificities). Interestingly, in the absence of I–HD binding sequences, ecoptic activity of the *lbl* enhancer did occur in the Slou-expressing VT1 muscle. We recently documented similar ectopic activity of the *lbl* enhancer when a sequence which is preferred by Slou and no other examined HDs is selectively mutated [5]. It remains unknown why this phenotype is I–HD-independent, but this observation might reflect the recently characterized requirement of the T box TF *optomotor-blind-related-gene 1* (*org-1*) gene acting upstream of Slou in regulating VT1 identity [26,46]. The synergistic association between NK HDs and Tbox TFs may also explain the weak activity of the de-repressed reporter [47,48] (Figure 4A’’).

### Transcriptional networks downstream of Hox and I–HD HD TFs

We have shown that Hox and I–HD TFs provide direct transcriptional input to both upstream and downstream components of the mesodermal gene regulatory network. These results confirm and extend previous genome-wide assessments of I–HD TF function, and confirm a contributory role of additional mesodermal HDs in regulating both upstream and downstream components of the myogenic gene regulatory network [5,13,22,26,43,49]. In addition, this study adds an additional layer of transcriptional specificity by documenting a critical and separate role played by I–HD TFs in regulating cell-specific mesodermal gene expression patterns.

A recent study documented on a global level the transcriptional targets of numerous Hox genes and confirmed transcriptional regulation of both upstream and downstream components [4]. In fact, the so-called realizator genes (downstream components) represented the most statistically over-represented Hox-responsive genes. A similar result was recently shown for Ubx-bound genomic regions in the haltere and leg in *Drosophila* [19]. Our results—which show integration of Hox TFs at the *mib2* and *Ndg* enhancers—confirm and extend these prior observations by establishing that the Hox genes are not simply regulating upstream components (signals and TFs) which then directly modulate their downstream targets. Rather, the results presented here document that Hox TFs themselves are regulating downstream components responsible for terminal cellular differentiation. These results prompt the consideration that the upstream targets of Hox TFs (for example, I-HDs and identity TFs having other classes of DNA binding domains) collaborate with the Hox TFs themselves to provide additional transcriptional response specificity to the downstream realizators in a feed-forward type of transcriptional network [2].

The TFs which direct myoblast differentiation, including the MyoD family of basic helix-loop-helix TFs, the Mef2 family of MADS-box TFs, and Six HD family members [50], have been conserved between *Drosophila* and vertebrates [50]. Differences in myogenesis do exist (e.g., the presence of muscle FCs that seed the formation of unique muscles have not been identified in mammals), which likely explains the failure to discover comparable individual muscle identity genes in mammals. However, there is extensive morphological and functional diversity among myogenic cells in vertebrates, including the existence of primary myofibers, secondary myofibers and satellite cells. In fact, recent work in mouse has shown that the Six subclass of HDs act as critical players in myofiber specialization through the selective activation of fast-type muscle genes [51,52]. As Six4 binding motifs were included in the analysis of I–HD function, this shows that Six4 HDs are playing a contributory role to regulating mesodermal gene expression programs, which is in agreement with preceding analyses of Six4 function in *Drosophila* [40]. Furthermore, in the present work, selective mutagenesis of Six binding sites in the *lbl* enhancer revealed their necessity for activating enhancer activity, which is consistent with the known role of Six4 in regulating *lbl* gene expression [40]. In addition, Six binding sites were required for the restriction of the *ap* enhancer to the correct myoblasts. Interestingly, Six4 binding sites were not necessary for appropriate activity of the *mib2* and *Ndg* enhancers, suggesting there might be differential requirements for Six4 in regulating upstream and downstream components of the mesodermal gene regulatory network.

### Specificity of HD function

In the present study, we have documented the critical and independent roles played by both I–HD and Hox TFs in directing appropriate mesodermal gene expression patterns. This raises the question of how transcriptional response specificity is achieved by these HD TFs, especially for those that primarily recognize the canonical TAAT core motif. One potential mechanism is HD target selectivity through the recognition of DNA sequences that are preferentially recognized by one HD [6,7], which we recently confirmed for the I–HD Slou in regulating myoblast gene expression [5]. It remains possible that the effects of other I–HD TFs (or Hox TFs) utilize a similar mechanism in the mesodermal enhancers under investigation here.

Another mechanism that needs to be considered is that the DNA specificity of Hox HDs is known to be modified by interactions with cofactors such as the PBC and MEIS subclasses of TALE HD proteins [3,53]. This mechanism is thought to raise the affinity of HD-DNA binding interactions and to create a longer binding site, with unique specificities generated by different HD-cofactor complexes [3,53,54]. Such a mechanism might explain the effects of Hox HDs in regulating mesodermal enhancers, although a similar explanation may not apply to I–HD TFs since there is currently no evidence that these Hox cofactors interact with *Drosophila* I–HD TFs. However, PBC proteins are thought to interact with similar classes of vertebrate TFs [55], raising the possibility that the functions of *Drosophila* I-HDs are influenced by TALE HD cofactors. Additionally, it has been recently shown that co-factor binding has the potential to change Hox DNA binding specificities, generating unique DNA binding preferences [45]. In the present study, we used the *in vitro* binding preferences for Ubx and AbdB, which were determined by PBM analysis in the absence of co-factors [5]. Nevertheless, our analysis of Hox monomers was sufficient to explain the Hox input to multiple mesodermal enhancers, which is in agreement with a previous study that documented transcriptional response specificity of HD proteins through the binding of multiple low affinity monomeric recognition sites [29]. In any event, the role of DNA binding preferences for Hox/co-factor complexes remains to be evaluated in the present system.

Finally, an additional layer of HD specificity could invoke the collaborative, combinatorial interactions of HD TFs with other genes, including TF heterodimerization [56,57], cooperative interactions with other cofactors [58], or formation of multi-protein complexes of signal-activated and tissue-restricted TFs having convergent effects on mesodermal gene expression [1,3]. In fact, recent work in other biological contexts has shown that Hox HDs work together with accessory factors such as other HD and forkhead domain proteins [59,60]. In agreement with this potential mechanism, we have recently defined a role for forkhead proteins in regulating the expression of *Ndg* and *ap* [26,35].

### Role of Tin in regulating mesodermal gene expression

The subdivision of the embryonic mesoderm in *Drosophila* requires the sequential deployment of a series of TFs, beginning with the expression of the basic helix-loop-helix TF Twist (Twi), which is required for the specification of the entire mesoderm [8]. Twi activates the expression of numerous additional TFs required for the subdivision of the mesoderm, including the NK-2 HD Tin [8]. Tin is initially expressed throughout the mesoderm but becomes restricted to the dorsal mesoderm, where its influence over the specification of the visceral and cardiac mesoderm, as well as dorsal somatic muscles, is pronounced [8,41]. However, the proper development of numerous ventral and lateral somatic muscles also requires Tin [41,42], suggesting that the early expression of Tin throughout the entire mesoderm affects genes directing ventro-lateral somatic myogenesis, a process that occurs after Tin expression disappears from myoblasts that develop in these locations. Interestingly, a genome-wide analysis of Tin binding suggested that these latter effects could be secondary to the activation of additional transcriptional components, which are required for somatic myogenesis in these tissues [15]. Such target genes include components of JAK/STAT signaling, as well as D-Six4. Here we have shown that the enhancers for the identity TFs *lbl* (which is active in one lateral myofiber) and *ap* (which is active in four lateral myofibers) receive I–HD input which includes contribution from Six4. This observation would help to explain the role of Tin in the specification of numerous ventro-lateral muscles, in spite of its expression being restricted to the dorsal mesoderm at the developmental time when these muscle FCs are specified.

Moreover, we have also shown that NK-2 binding sequences are required for the proper activity of *lbl*, *mib2* and *Ndg* enhancers, all of which are active in ventro-lateral cells that develop after Tin expression becomes restricted to the dorsal mesoderm. This finding suggests that these enhancers are receiving input from Tin, which is in agreement with the discovery of in vivo Tin binding to regulatory regions near muscle identity genes [15]. Furthermore, we have shown that the presence of Tin binding sites is a good predictor of muscle FC gene activity [22,26]. Finally, an examination of ChIP data revealed that Tin was the most enriched TF within a large set of mesodermal enhancers at early developmental stages when Tin is expressed ubiquitously throughout the mesoderm (S. S. G, L. A. Barerra, M. Porsch, A. Aboukhalil, P. W. Estep 3rd, A. Vedenko, A. Palagi, Y. Kim, X. Zhu, B. W. B., C. E. Gamble, A. Iagovitina, A.M. M, and M. L. B., manuscript submitted). Collectively, these data suggest that Tin might serve as a pioneer factor that marks enhancers for activity that only occurs at later developmental stages, that is, after the TF itself is no longer present in the cells in which the enhancer of interest becomes functional [61]. Thus, Tin possesses multiple roles throughout myogenesis along the entire dorso-ventral axis, including directly targeting additional muscle identity TFs. These TFs then function together in a feed-forward loop to target other components that are required for the proper specification and differentiation of individual somatic muscles [1,2,62].

## Conclusions

Here we utilized the complete spectrum of DNA binding preferences for a diverse set of mesodermally-expressed HDs to examine whether Hox and I–HD TFs independently contribute to cell-specific gene expression programs, a problem that has not been addressed before since, until recently, the relevant information for designing the requisite experiments was not available. Our results show that both subclasses of HD are separately integrated by the unique combinations of DNA binding motifs that are located within multiple mesodermal enhancers that control the expression of genes representing both upstream and downstream components of the mesodermal gene regulatory network. In addition, we describe a role for NK-2 HD binding sites in regulating gene expression in mesodermal cells located throughout the embryo, which may provide a potential explanation for how Tin contributes to enhancer activity in myoblasts at developmental times in which it is no longer expressed. Similar applications of the approach that we have employed here could be used to uncouple the contribution of individual TF family members that have similar DNA binding profiles in other developmental systems.

## Supporting Information

Figure S1 Targeted mutagenesis of HD, I–HD, Hox and NK binding sequences in the *ap* enhancer. E-score (y-axis) binding profiles of the indicated HD TFs to the wild-type *ap* enhancer and versions in which all HD, I–HD, Hox or Tin binding sites are mutated. The horizontal black line represents a threshold binding E-score of 0.31 below which binding is not considered significant, and was chosen as described in the Materials and Methods [5].
(TIF)

Figure S2 Targeted mutagenesis of HD, I–HD, Hox and NK binding sequences in the *lbl* enhancer. E-score (y-axis) binding profiles of the indicated HD TFs to the wild-type *lbl* enhancer and versions in which all HD, I–HD, Hox or Tin binding sites are mutated. The horizontal black line represents a threshold binding E-score of 0.31 below which binding is not considered significant, and was chosen as described in the Materials and Methods [5].
(TIF)

Figure S3 Targeted mutagenesis of HD, I–HD, Hox and NK binding sequences in the *mib2* enhancer. E-score (y-axis) binding profiles of the indicated HD TFs to the wild-type *mib2* enhancer and versions in which all HD, I–HD, Hox or Tin binding sites are mutated. The horizontal black line represents a threshold binding E-score of 0.31 below which binding is not considered significant, and was chosen as described in the Materials and Methods [5].
(TIF)

Figure S4 Targeted mutagenesis of HD, I–HD, Hox and NK binding sequences in the *Ndg* enhancer. E-score (y-axis) binding profiles of the indicated HD TFs to the wild-type *Ndg* enhancer and versions in which all HD, I–HD, Hox or Tin binding sites are mutated. The horizontal black line represents a threshold binding E-score of 0.31 below which binding is not considered significant, and was chosen as described in the Materials and Methods [5].
(TIF)

Table S1 PBM results and position-weight matrices for Caup (XLSX)

Table S2 Wild-type and mutant enhancer sequences considered in this study as well as versions of the wild-type enhancers in which the different HD subclasses are highlighted. Detailed E-score information relevant to the wild-type and mutant sequences shown for the enhancers in Table S2 can be found in Busser et al. [5] and in Table S1 for Caup.
(XLS)
