# Supplementary figures and images for: Contribution of Distinct Homeodomain DNA Binding Specificities to Drosophila Embryonic Mesodermal Cell-Specific Gene Expression Programs
Source: PLoS One. 2013 Jul 26;8(7):e69385. doi: 10.1371/journal.pone.0069385 (PMC3724861; doi:10.1371/journal.pone.0069385)

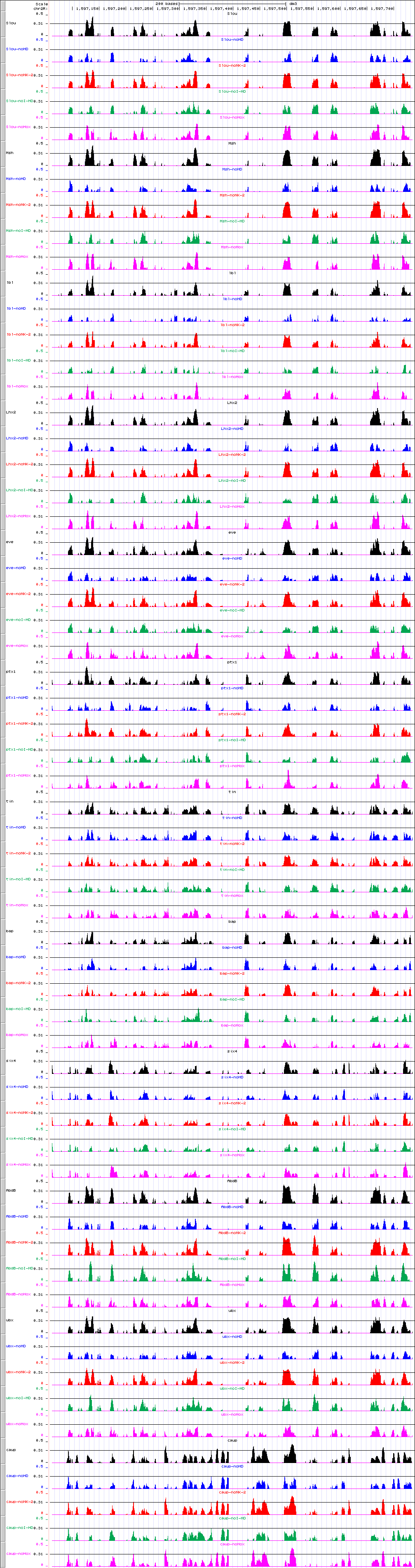

Supplement: Figure S1 — E-score (y-axis) binding profiles of the indicated HD TFs to the wild-type ap enhancer and versions in which all HD, I–HD, Hox or Tin binding sites are mutated. The horizontal black line represents a threshold binding E-score of 0.31 below which binding is not considered significant, and was chosen as described in the Materials and Methods [5]. (TIF) [file pone.0069385.s001.tif]

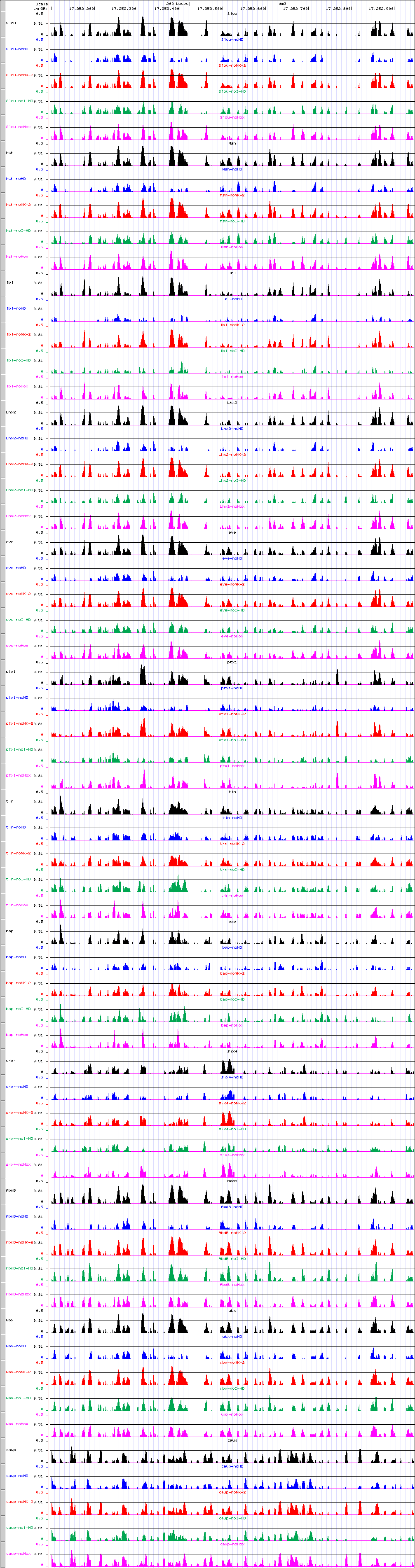

Supplement: Figure S2 — E-score (y-axis) binding profiles of the indicated HD TFs to the wild-type lbl enhancer and versions in which all HD, I–HD, Hox or Tin binding sites are mutated. The horizontal black line represents a threshold binding E-score of 0.31 below which binding is not considered significant, and was chosen as described in the Materials and Methods [5]. (TIF) [file pone.0069385.s002.tif]

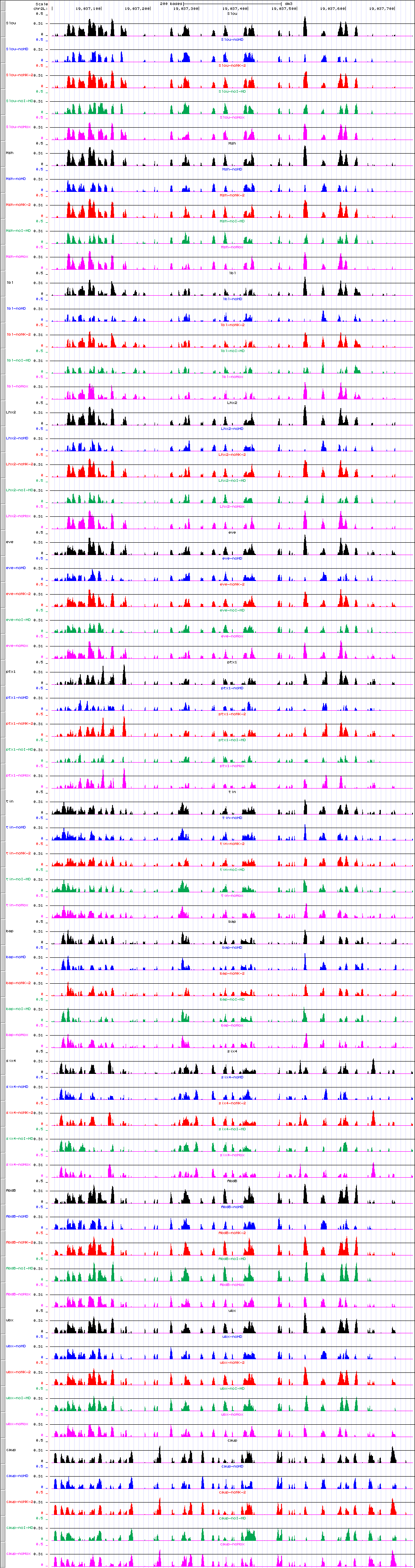

Supplement: Figure S3 — E-score (y-axis) binding profiles of the indicated HD TFs to the wild-type mib2 enhancer and versions in which all HD, I–HD, Hox or Tin binding sites are mutated. The horizontal black line represents a threshold binding E-score of 0.31 below which binding is not considered significant, and was chosen as described in the Materials and Methods [5]. (TIF) [file pone.0069385.s003.tif]

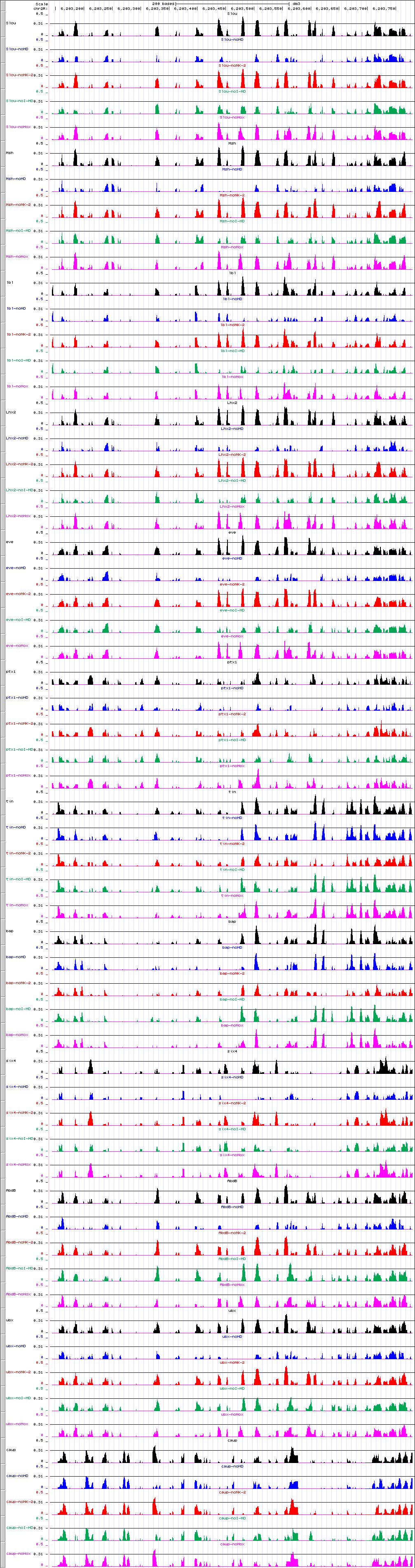

Supplement: Figure S4 — E-score (y-axis) binding profiles of the indicated HD TFs to the wild-type Ndg enhancer and versions in which all HD, I–HD, Hox or Tin binding sites are mutated. The horizontal black line represents a threshold binding E-score of 0.31 below which binding is not considered significant, and was chosen as described in the Materials and Methods [5]. (TIF) [file pone.0069385.s004.tif]
